# Academy of Medicine—Deleterious Effects of Swill-Milk

**Published:** 1859-07

**Authors:** 


*Academy of Medicine—Deleterious Effects of Swill-Milk.*—At the late regular meeting of the Academy of Medicine, a report was presented on behalf of the committee to whom had been referred the matter of preparing a reply to Mayor TIEMANN's inquiry as to the effect on the human system of the milk of swill-fed cows. The report was read by Dr. SAMUEL R. PERRY. Accompanying it was an elaborate statement of the investigations in reference to this subject, in which Dr. S. has been personally engaged for several months, and which he has pursued with great intelligence and assiduity. This statement shows that the condition in which swill-fed cows are kept gives undeniable evidence of the poisonous effects of their milk. Their stables, instead of giving forth the healthy aroma of country fed cattle, are pervaded by a sickening stench. The cows themselves are the victims of disease engendered by the food on which they live. Eighteen pounds of corn and a little straw given daily, is abundant to keep a cow in good flesh. But to obtain the same quantity of nutritive element from swill, 130 gallons of it must be taken daily; that is, 40 gallons to supply the nitrogen, 30 to supply the oleaginous matter, and 60 to supply the hydro-carbonate. In taking so much swill a cow will consume daily one quart of vinegar. In the course of the Doctor's experiments he found that this milk gave a strong acid reaction; and, from series of results derived from personal observation, he had dicovered that the milk of unhealthy women, living in damp cellars and eating bad food, or habitually intemperate, exhibited the same characteristic. Having thus established *prima facie*

his case against swill-milk, the Dr. proceeded to furnish instances in which young children, born of healthy parents and healthy when born, or brought in a healthy condition from the country to the city, had incurred disease by the use of swill-milk and recovered when it was withheld from them. He exhibited numerous analyses of different kinds of milk and various microscopic drawings of swill-milk, showing in every case conferves and sporads. These were examined with much interest. The academy resolved to send an engrossed copy of the report to Mayor TIEMANN, and return a vote of thanks to the committee, who were then discharged.—*N. Y. Times.*

